# Supplementary figures and images for: Neonatal unit human resources: coverage for six cadres and trends for staff-to-baby ratios in 65 neonatal units implementing with NEST360 in Kenya, Malawi, Nigeria, and Tanzania
Source: Hum Resour Health. 2025 Nov 12;23:64. doi: 10.1186/s12960-025-01031-1 (PMC12613486; doi:10.1186/s12960-025-01031-1)

## Day of visit

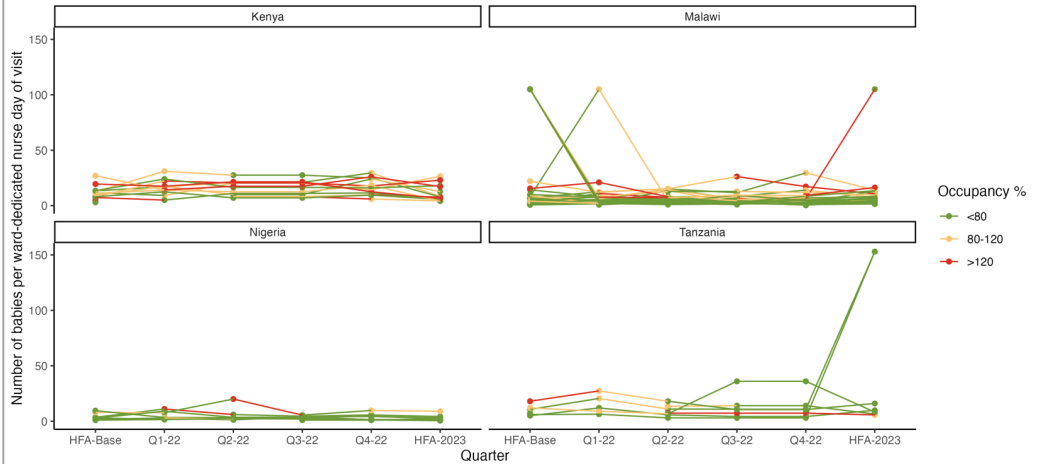

## Night before visit

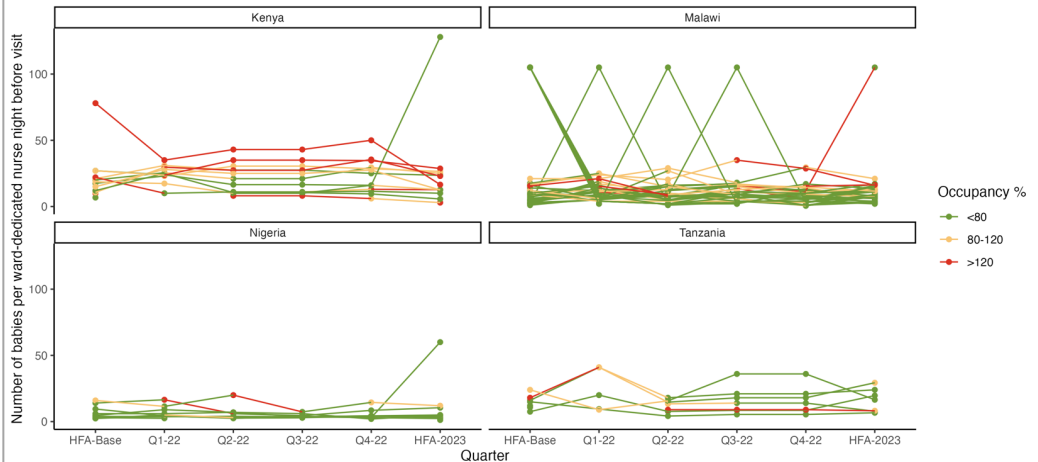

Supplement: Supplementary file 2 — Additional file 2: Ward-specific nurse-to-baby ratios on day of and night before the visit with bed occupancy over time at 65 neonatal units in Kenya, Malawi, Nigeria, and Tanzania. Q; Quarter, HFA; Health Facility Assessment, Base; Baseline. [file 12960_2025_1031_MOESM2_ESM.pdf]

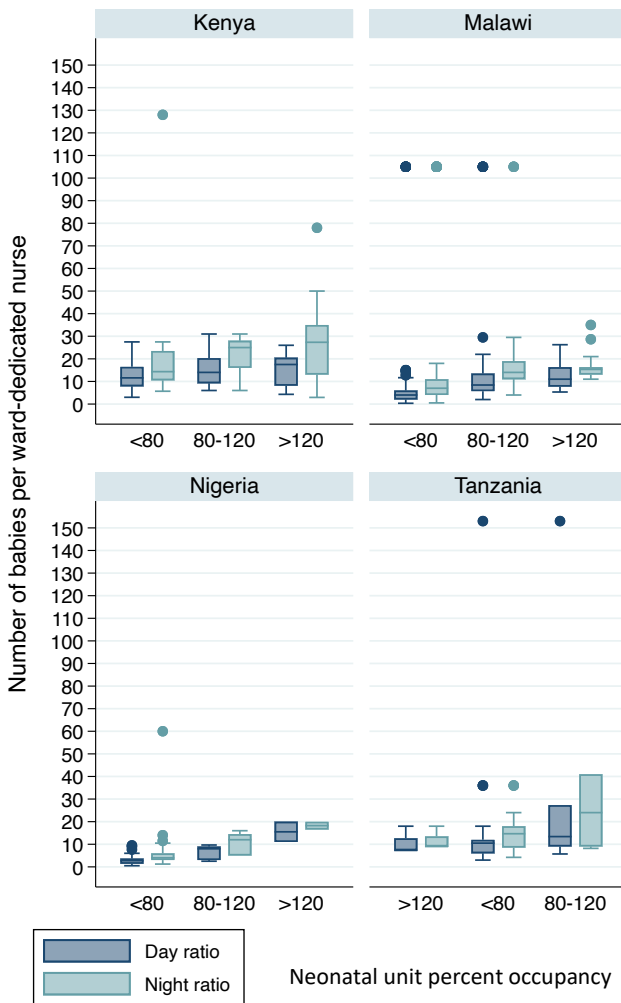

Supplement: Supplementary file 3 — Additional file 3: Boxplot of neonatal ward-specific nurse-to-baby ratios by occupancy and by country over time at 65 neonatal units in Kenya, Malawi, Nigeria, and Tanzania. [file 12960_2025_1031_MOESM3_ESM.pdf]
