# Supplementary material for: Neonatal unit human resources: coverage for six cadres and trends for staff-to-baby ratios in 65 neonatal units implementing with NEST360 in Kenya, Malawi, Nigeria, and Tanzania
Source: Hum Resour Health. 2025 Nov 12;23:64. doi: 10.1186/s12960-025-01031-1 (PMC12613486; doi:10.1186/s12960-025-01031-1)
Supplement: Supplementary file 4 — Additional file 4: Clinical and non-clinical staff coverage and skill-mix at 65 neonatal units in Kenya, Malawi, Nigeria, and Tanzania. [file 12960_2025_1031_MOESM4_ESM.docx]

**Additional file 4**: *Clinical and non-clinical staff coverage and skill-mix at 65 neonatal units in Kenya, Malawi, Nigeria, and Tanzania*.

|  | Overall | Malawi | Kenya | Tanzania | Nigeria |
| --- | --- | --- | --- | --- | --- |
| Neonatal Units | 65 | 36 | 13 | 7 | 9 |
| Nursing coverage and skill-mix | | | | | |
| ≥1 nurse (any) providing ward-specific care on neonatal unit (employed)^±^ | | | | | |
| HFA – Baseline (2019-21) | 57 (88) | 28 (78) | 13 (100) | 7 (100) | 9 (100) |
| HFA - 2023 | 62 (95) | 35 (97) | 13 (100) | 5 (71) | 9 (100) |
| ≥1 specialist nurse providing ward-specific care on neonatal unit (employed)^±^ | | | | | |
| HFA – Baseline (2019-21) | 20 (31) | 1 (3) | 10 (77) | 1 (14) | 8 (89) |
| HFA - 2023 | 22 (34) | 6 (17) | 9 (69) | 0 (0) | 7 (78) |
| Percentage of all employed nurses that are specialist nurses (out of any nurses providing ward-specific care on neonatal unit)^±^ | | | | | |
| HFA – Baseline (2019-21) | 0% (0-20%) | 0% (0-0%) | 22% (17-33%) | 0% (0-0%) | 50% (17-50%) |
| HFA - 2023 | 0% (0-25%) | 0% (0-0%) | 33% (0-54%) | 0% (0-0%) | 24% (17-50%) |
| Percentage of all nurses assigned to neonatal unit that are locum | | | | | |
| HFA – Baseline (2019-21)* | 4% (0-15%) | 9% (3-16%) | 0% (0-2%) | 17% (0-55%) | 0% (0-0%) |
| HFA - 2023 | 0% (0-11%) | 0% (0-12%) | 0% (0-11%) | 11% (4-40%) | 0% (0-0%) |
| Doctor and clinical officer coverage and skill-mix | | | | | |
| ≥1 doctor providing care to the neonatal unit (day) | | | | | |
| HFA – Baseline (2019-21) | 35 (54) | 6 (17) | 13 (100) | 7 (100) | 9 (100) |
| HFA - 2023 | 36 (55) | 8 (22) | 13 (100) | 7 (100) | 8 (89) |
| ≥1 doctor providing care to the neonatal unit (night) | | | | | |
| HFA – Baseline (2019-21) | 19 (29) | 1 (3) | 3 (23) | 6 (86) | 9 (100) |
| HFA - 2023 | 18 (28) | 0 (0) | 4 (31) | 6 (86) | 8 (89) |
| ≥1 doctor providing care to the neonatal unit (day) + on call | | | | | |
| HFA – Baseline (2019-21) | 35 (54) | 6 (17) | 13 (100) | 7 (100) | 9 (100) |
| HFA - 2023 | 36 (55) | 8 (22) | 13 (100) | 7 (100) | 8 (89) |
| ≥1 doctor providing care to the neonatal unit (night) + on call | | | | | |
| HFA – Baseline (2019-21) | 20 (31) | 1 (3) | 4 (31) | 6 (86) | 9 (100) |
| HFA - 2023 | 18 (28) | 0 (0) | 4 (31) | 6 (86) | 8 (89) |
| ≥1 specialist doctor assigned to neonatal unit (employed) | | | | | |
| HFA – Baseline (2019-21) | 32 (49) | 3 (8) | 13 (100) | 7 (100) | 9 (100) |
| HFA - 2023 | 33 (51) | 6 (17) | 13 (100) | 7 (100) | 7 (78) |
| ≥1 specialist doctor providing ward-specific care on neonatal unit (employed) | | | | | |
| HFA – Baseline (2019-21) | 20 (31) | 1 (3) | 5 (38) | 5 (71) | 9 (100) |
| HFA - 2023 | 30 (46) | 4 (11) | 13 (100) | 6 (86) | 7 (78) |
| Biomedical technician/engineer coverage and skill-mix | | | | | |
| ≥1 employed biomedical technician/engineer (assigned to one or multiple facilities) | | | | | |
| HFA – Baseline (2019-21) | 45 (69) | 19 (51) | 11 (85) | 7 (100) | 8 (89) |
| HFA - 2023 | 56 (86) | 27 (73) | 13 (100) | 7 (100) | 9 (100) |
| ≥1 employed biomedical technician/engineer (hospital-specific) | | | | | |
| HFA – Baseline (2019-21)* | 36 (65) | 12 (43) | 10 (91) | 7 (100) | 7 (78) |
| HFA - 2023 | 51 (78) | 25 (69) | 10 (77) | 7 (100) | 9 (100) |
| Number of biomedical technicians employed | | | | | |
| HFA – Baseline (2019-21)* | 1 (0-2) | 1 (0-1) | 1 (0-5) | 3 (0-5) | 2 (0-6) |
| HFA - 2023 | 1 (1-4) | 1 (0-1) | 7 (5-9) | 5 (2-6) | 0 (0-4) |
| Number of engineers employed | | | | | |
| HFA – Baseline (2019-21)* | 0 (0-2) | 0 (0-0) | 5 (2-9) | 1 (0-1) | 3 (1-5) |
| HFA - 2023 | 0 (0-1) | 0 (0-0) | 1 (0-1) | 1 (1-3) | 3 (2-4) |
| Neonatal unit data clerk coverage | | | | | |
| ≥1 data clerk assigned to the neonatal unit | | | | | |
| HFA – Baseline (2019-21) | 32 (49) | 21 (58) | 7 (54) | 1 (14) | 4 (44) |
| HFA - 2023 | 58 (89) | 35 (97) | 13 (100) | 4 (57) | 6 (67) |
| Laboratory coverage | | | | | |
| Laboratory coverage 24 hours per day | | | | | |
| HFA – Baseline (2019-21)* | 25 (45) | 5 (18) | 10 (91) | 6 (86) | 4 (44) |
| HFA - 2023 | 31 (48) | 8 (22) | 13 (100) | 6 (86) | 4 (44) |
| Laboratory coverage (weekday and weekend days) | | | | | |
| HFA – Baseline (2019-21)* | 49 (89) | 26 (93) | 11 (100) | 6 (86) | 6 (67) |
| HFA – 2023 | 47 (72) | 23 (64) | 13 (100) | 6 (86) | 5 (56) |
| Laboratory coverage (weekday and weekend nights) | | | | | |
| HFA – Baseline (2019-21)* | 26 (47) | 6 (21) | 10 (91) | 6 (86) | 4 (44) |
| HFA - 2023 | 31 (48) | 8 (22) | 13 (100) | 6 (86) | 4 (44) |

**Legend**:

- Specialist doctors include paediatricians and neonatologists.
- Specialist nurses include nurses with paediatric or neonatal training as relevant for country guidelines.
- Cells show number of neonatal units (percentage) for categorical variables, or median (IQR) for numeric variables.
- *Missing data for 10 neonatal units that completed different version of baseline HFA tool (n=28 for Malawi, n=11 for Kenya). Total denominator includes 55 neonatal units.
- ^±^Shows daytime staffing numbers only.
- HFA: Health Facility Assessment
